# Sotatercept Use in a Patient with Pulmonary Arterial Hypertension Undergoing Lung Transplantation

**DOI:** 10.1016/j.jhlto.2025.100213

**Published:** 2025-01-14

**Authors:** Justin P. Rosenheck, Kashika Goyal, Tara Fallah, Pamela Burcham, Kukbin Choi, Matthew Henn, Elie Homsy, Scott Visovatti, Veronica Franco

**Affiliations:** a Ohio State University, Department of Internal Medicine, Division of Pulmonary Critical Care and Sleep Medicine, Davis Heart and Lung Institute, College of Medicine, The Ohio State University Wexner Medical Center, Columbus, OH; b Ohio State University, College of Pharmacy, 217 Lloyd M. Parks Hall, 500 West 12th Ave, Columbus OH 43210; c Ohio State University, Department of Surgery, Division of Cardiac Surgery, 410 W. 10th Ave, Columbus OH 43210; d Ohio State University, Department of Internal Medicine, Division of Cardiovascular Medicine, 452 W 10th Ave, Columbus, OH 43210

**Keywords:** Pulmonary Arterial Hypertension, PAH, Group 1, Lung Transplant, Sotatercept

## Abstract

Pulmonary arterial hypertension (PAH) is one of the common indications for lung transplantation. Sotatercept is a new medication with a novel mechanism of action and was recently approved for the treatment of PAH. Sotatercept is associated with significant adverse effects including thrombocytopenia and erythrocytosis which can impact outcomes of lung transplantation. This is the first described case of a patient undergoing lung transplantation while receiving sotatercept for PAH.

## Intro

Lung transplantation is indicated for patients with severe, life-threatening pulmonary diseases, including pulmonary arterial hypertension (PAH) when the disease course is not improved with optimal medical care. For patients with PAH, this includes use of medications targeting relevant pathways in the pathogenesis of pulmonary hypertension, including the prostacyclin, endothelin and nitric oxide pathways.^1^ Sotatercept is a first-in-class fusion protein indicated for the treatment of patients with group 1 pulmonary hypertension. Sotatercept features an activin receptor type IIA (ActRIIA), which functions as a ligand trap by binding to members of the transforming growth factor (TGF) B family and restores the balance between pro-proliferative and anti-proliferative signals. Sotatercept has been demonstrated in animal models to inhibit pulmonary endothelial and smooth muscle proliferation, promote apoptosis and lead to reverse remodeling and improved patency of pulmonary arterial vessels.^2^, ^3^

The STELLAR trial evaluated the role of sotatercept versus placebo in patients with functional class (FC) II or III PAH. The primary end point of this study demonstrated that sotatercept significantly improved six-minute walk distance at 24 weeks in patients on appropriate background therapy.^3^ Important secondary endpoints were also achieved at 24 weeks, including improvements in pulmonary vascular resistance (PVR), NT-proBNP levels, FC, and importantly increased time elapsed before either death or clinical worsening. These secondary endpoints are especially important to consider in lung transplant candidates, as time spent on the transplant waiting list is a high-risk period for clinical deterioration, or death. Population health modeling suggests sotatercept may decrease the incidence of PAH-related hospitalizations and the need for lung or heart-lung transplantation.^4^ However, sotatercept also has significant adverse effects (AE) that may impact its use in patients listed for lung transplantation.^3^ Bleeding (primarily epistaxis and gingival bleeding), thrombocytopenia and telangiectasias were seen more frequently in the treatment than the placebo group in the STELLAR trial. Additional nuance is introduced for patients awaiting lung transplantation when considering the long half-life of sotatercept. The risk of adverse effects needs to be weighed carefully against the potential benefits in patients listed for lung transplantation. Here, we describe the first case of a patient who underwent lung transplantation, while receiving sotatercept.

## Case

**Figure 1:**
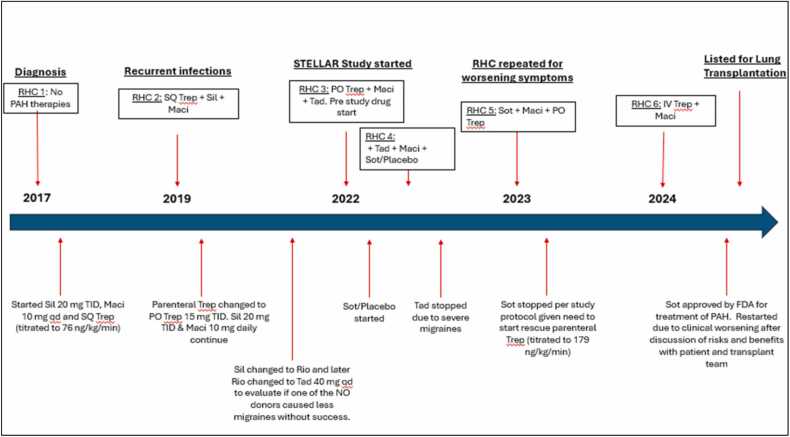
Timeline of Therapies and Hemodynamic Assessments. Abbreviations: Maci: Macitentan; Rio: Riociguat; Sil: Sildanefil; Sot: Sotatercept; Tad: Tadalafil; Trep: treprostinil; IV: Intravenous; qd: every day; PO: Per Oral; SQ: Subcutaneous; TID: three times daily.

The patient is a 61-year-old female with a history of BMPR-2 negative idiopathic PAH diagnosed in 2017 and well controlled systemic hypertension. Her care was delivered through a specialty clinic and early PAH therapy included parenteral treprostinil, sildenafil and macitentan (hemodynamics and medications detailed in Table 1 and Figure 1). Intravenous and then subcutaneous treprostinil were ultimately switched to oral treprostinil at equivalent dosing given concerns for recurrent infusion site infections. Due to recurrent migraines, sildenafil was switched to tadalafil. While on stable background therapy of oral prostacyclin, macitentan and tadalafil, this patient was evaluated and enrolled in the STELLAR trial and received either sotatercept or placebo.

**Table 1:** Pulmonary Hemodynamic Values

	RA Pressure mmHg	PA Pressures (Mean) mmHg	PA Occlusion Pressure mmHg	PA Sat	Fick CO / CI	PVR (Wood Units)	Echo Findings	PAH Specific Medications
RHC 1: 11/2018	7	71/37 (48)	28 (EDP 8)	59%	4.0/2.3	10	RV: Severe dysfunction	None
RHC 2: 12/2019	6	56/23 (36)	9	76%	5.5/3.1	4.9	RV: Enlarged, Normal EF	SQ Trep 76 ng/kg/min + Sil 20mg+Maci 10 mg
RHC 3: 08/2021	9	76/27 (48)	10	77%	7.1/4.2	5.2	RV: Enlarged, Normal EF	PO Trep 15 mg TID + Sil 20 mg q + Maci 10 mg
RHC 4: 02/2022	5	37/19 (25)	7	60%	4.0/2.4	4.4		PO Trep 15 mg TID +Tad 40 mg + Maci 10 mg + Sot or Placebo
RHC 5: 6/2023	5	49/23 (34)	7	58%	3.1/1.8	8.7	LV: D-shaped RV: Severely enlarged, mild dysfunction	PO Trep 15 TID + Maci 10 mg + Sot
RHC 6: 3/2024	14	100/49 (69)	13		4.1/2.4	13.7		IV Trep 179 ng/kg/min + Maci 10 mg qd

Abbreviations: CI: Cardiac index; CO: Cardiac output; HR: EDP: End diastolic pressure; EF: Ejection Fraction; Heart rate; LV: left ventricle; PA: Pulmonary artery; PVR: Pulmonary vascular resistance; RA: Right atrium; RV: Right ventricle; SBP: systolic blood pressure; SVR: systemic vascular resistance; Maci: Macitentan; Sil: Sildanefil; Sot: Sotatercept; Tad: Tadalafil; Trep: Treprostinil; IV: Intravenous; PO: Per Oral SQ: Subcutaneous

**Figure 2:**
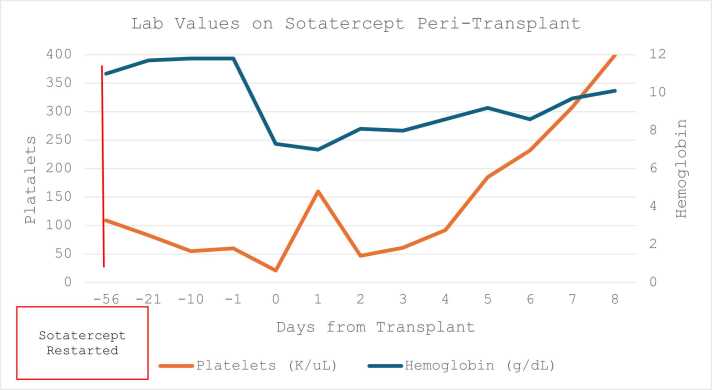
Lab Values on Sotatercept Peri-Transplant.

During the study period, she developed mild erythrocytosis with a peak Hb of 16.5 g/dL and mild-moderate thrombocytopenia with a nadir platelet count of 95 K/uL (Figure 2). She experienced one episode each of mild epistaxis and gingival bleeding which led to a dose reduction in study drug/placebo. A repeat right heart catheterization demonstrated marked improvements in mean pulmonary artery (PA) pressures. Per protocol, study drug was continued for one year before she was switched to open label sotatercept. During the open label extension, tadalafil was discontinued for severe, persistent migraines. She subsequently developed worsening hemodynamics and FC and thus IV treprostinil was restarted. Per study protocol, sotatercept was discontinued given the need to reinstitute rescue treprostinil. Due to worsening pulmonary hemodynamics and symptomatology at this time, a decision was made to begin workup for lung transplantation. Despite titration of treprostinil to 179 ng/kg/min, her PAH continued to progress with worsening symptoms to FC IV, PVR of 13.7, mean PA pressure of 69 mmHg, and a REVEAL 2.0 score of 11.

With worsening symptoms and hemodynamics, reinstitution of sotatercept was discussed with the patient and lung transplant team with a focus on known hematologic risks including thrombocytopenia and erythrocytosis. A decision was made to restart this drug while on the waitlist and continue long as platelets remained above 75 K/uL. Sotatercept was restarted at 0.3mg/kg, with subsequent doses of 0.7 mg/kg. Following reinstitution of sotatercept, her dyspnea improved, FC improved to class III, though REVEAL 2.0 score remained high risk at 10. Thrombocytopenia to 55K/uL developed and a dose was held. After approximately six weeks on the waitlist, and four weeks after her last dose of sotatercept, an appropriate donor was identified. On admission, hemoglobin was 11.8 g/dL and platelet count was 60K/uL. Transplant was performed via bilateral thoracosternotomy on VA-ECMO. Intra-operatively, four units of packed red blood cells (RBCs), two units of platelets, four units of cryoprecipitate and 5 units of plasma were administered. Point of care thromboelastography was used to direct appropriate administration of hemostatic products. She developed reperfusion edema at the conclusion of this case, thus bifemoral VV-ECMO was initiated to support her recovery in the intensive care unit.

The patient’s course was complicated by grade 3 primary graft dysfunction (PGD) and postoperative bleeding secondary to a right internal mammary artery (IMA) laceration. 4 additional units of packed RBCs were given on POD 1 before the patient returned to the OR for definitive surgical hemostasis, with no additional transfusions administered thereafter. VV-ECMO was discontinued on post-operative day (POD) 3, and the patient was extubated the following day, with a rapid recovery to room air oxygen. Her subsequent hospital course was uneventful, and she was discharged to independent housing on POD 12. At discharge, hematological abnormalities havd fully resolved. Routine surveillance transbronchial biopsies at 1- and 3-months post transplantation showed no evidence of acute cellular rejection (ACR). At 3 months post-transplantation, this patient remains on standard institutional doses of maintenance tacrolimus, mycophenolate mofetil and prednisone. There is no evidence of ACR or antibody mediated rejection and she has excellent allograft function.

## Discussion

We report the first case of a patient who underwent successful lung transplantation while receiving sotatercept, a novel agent recently approved for patients with PAH. This report is timely and relevant as lung transplant and pulmonary hypertension providers are increasingly likely to face this situation. At present, no guidelines exist for the management of this medication in transplant candidates.

Sotatercept has a long half-life of elimination of approximately 24 days and a time to peak effect of around 7 days.^5^ It could be expected that waitlisted lung transplant candidates receiving sotatercept may experience the effects of this drug at the time of transplantation. In the STELLAR trial, adverse events included severe thrombocytopenia (platelets <50K/uL) in 6.1%, increases in hemoglobin in 5.5%, and bleeding events in 21.5% including epistaxis in 12.3%.^3^ These risks need to be balanced against the expected benefits of this new therapeutic option.^6^

In this case, the patient demonstrated significant clinical and hemodynamic decline when sotatercept was discontinued, with subsequent clinical improvement when resumed. Given known hematologic risks, our team opted for close monitoring of hematologic complications, with a plan to manage peri-operative bleeding with blood product transfusion. The use of excess blood products has been shown to increase the risk of PGD,^7^ and development of de novo donor specific antibodies,^8^ however our team felt that the risk of discontinuing sotatercept again in this patient outweighed these potential risks. This patient ultimately did require multiple transfusions, although not an exceptional amount compared to other cases at our institution.

There are many agents currently used in advanced lung diseases which may confer additional risk peri-transplant. In some, the putative mechanisms of these agents dictate their use or discontinuation on the transplant waitlist. In others, clinical experience functions as a guide.^9^, ^10^, ^11^ In all cases, risks must be balanced with the benefit of forestalling additional lung function decline. In this case, sotatercept stabilized our patient’s clinical status, allowing her to remain on the lung transplant wait list. We felt that a mandated a period of drug wash-out, followed by a potentially lengthy period on the waitlist, could have led to further decline of her pulmonary hypertension, exposing her to significant risk.

This case emphasizes the need for clear guidelines on when and how to safely continue therapies in the transplant setting. Sotatercept is a potentially valuable agent that can serve as a bridge to lung transplantation in PAH patients at high risk of disease progression. An attempt to discontinue sotatercept was made in this patient, however her subsequent clinical and hemodynamic decline demonstrates that its effects may not persist long term without continuous therapy. Reinstitution of sotatercept while on the waitlist required balancing potential hematologic complications against the risk of PAH progression. Close hematologic and clinical monitoring is necessary for waitlisted patients on this medication and alterations to dosing and administration schedule can mitigate known adverse effects. Teams should be aware of potential risks related to transfusion of blood products in transplant patients. In this first reported case of a patient with PAH treated with sotatercept undergoing lung transplantation, we have demonstrated that this medication can be safely continued in patients listed for lung transplantation.

## Funding

This research did not receive any specific grant from funding agencies in the public, commercial, or not-for-profit sectors.

## Declaration of Competing Interest

The authors declare the following financial interests/personal relationships which may be considered as potential competing interests: Veronica Franco reports a relationship with Merck & Co Inc that includes: consulting or advisory. Veronica Franco reports a relationship with Gossamer Bio Inc that includes: consulting or advisory. If there are other authors, they declare that they have no known competing financial interests or personal relationships that could have appeared to influence the work reported in this paper.
