# Corrigendum to ‘‘Sotatercept use in a patient with pulmonary arterial hypertension undergoing lung transplantation’’ [JHLT Open (2025) 100213]

**DOI:** 10.1016/j.jhlto.2025.100430

**Published:** 2025-11-05

**Authors:** Justin P. Rosenheck, Kashika Goyal, Tara Fallah, Pamela Burcham, Kukbin Choi, Matthew Henn, Elie Homsy, Scott Visovatti, Veronica Franco

**Affiliations:** a Ohio State University, Department of Internal Medicine, Division of Pulmonary Critical Care and Sleep Medicine, Davis Heart and Lung Institute, College of Medicine, The Ohio State University Wexner Medical Center, Columbus, Ohio; b Ohio State University, College of Pharmacy, Columbus, Ohio; c Ohio State University, Department of Surgery, Division of Cardiac Surgery, Columbus, Ohio; d Ohio State University, Department of Internal Medicine, Division of Cardiovascular Medicine, Columbus, Ohio

The authors regret that the listed patient consent statement was not included in the article.

## Consent

The patient has supplied informed written consent for this case study to be submitted.

The authors would like to apologize for any inconvenience caused.

